# Electrical stimulation of neuroretinas with 3D pyrolytic carbon electrodes

**DOI:** 10.1007/s10544-024-00729-8

**Published:** 2025-02-11

**Authors:** Pratik Kusumanchi, Jesper Guldsmed Madsen, Toke Bek, Stephan Sylvest Keller, Rasmus Schmidt Davidsen

**Affiliations:** 1 National Centre for Nano Fabrication and Characterization (DTU Nanolab), Technical University of Denmark, Kongens Lyngby, Denmark; 2 Department of Ophthalmology, Aarhus University Hospital, Aarhus, Denmark; 3 Department of Electrical and Computer Engineering, Aarhus University, Aarhus, Denmark

**Keywords:** Retinal prosthesis, Electrical stimulation, Pyrolytic carbon

## Abstract

Retinal prosthesis has been one of the medical strategies aimed at restoring some degree of vision for patients affected by retinal degenerative diseases, such as Retinitis Pigmentosa (RP) and age-related macular degeneration (AMD), which are leading causes of irreversible visual loss. In retinal prosthesis, electrical pulses are typically delivered to the retinal neurons via electrodes on the surface of the implant. In this work, we fabricated 3D carbon pillar electrodes by pyrolysis of SU-8 structures defined photolithographically on Si wafers. We then measured compound action potentials induced in porcine neuroretinas stimulated with electrical pulses. The recorded spikes were validated to be biological in origin by adding the voltage-gated sodium-channel blocking agent tetrodotoxin. The minimum threshold voltage needed to effectively stimulate retinal cells, such as retinal ganglion cells, with 3D electrodes was analyzed through systematic investigation of the spike rate and amplitudes as a function of stimulation voltage. 3D electrodes significantly increased spike rate and amplitudes above spontaneous activity in the tissue during stimulation and outperformed the 2D counterpart, both in terms of spike rate and amplitude. Our results indicate a threshold voltage range of 500-600 mV for 1 ms pulses at a frequency of 10 Hz above which a significant increase in spike count was observed. Furthermore, we report an order of magnitude increase in peak-to-peak amplitude for evoked spikes (> 3 mV), compared to spontaneous spikes (∼ 200 µV). Based on numerical integration, we estimate the area under the curve to be ~14 times larger in evoked compound action potentials compared to spontaneous activity. This indicates the relative increase in number of contributing cells to the compound action potential. At a stimulation voltage of 600 mV the spike rate for 3D electrodes was above 10 spikes/channel/s. We hypothesize that the significant difference between 2D and 3D electrodes is not only caused by the higher active electrode surface area of the 3D micropillar electrodes, but also by more intricate contact and interaction with the inner cell layers of the retinal tissue. Our findings indicate that 3D carbon micropillar electrodes are promising for electrical stimulation of the retina.

**Supplementary Information:**

The online version contains supplementary material available at 10.1007/s10544-024-00729-8.

## Introduction

Retinal degenerative diseases, such as Retinitis Pigmentosa (RP) and Age-related Macular Degeneration (AMD), are leading causes of irreversible visual loss. Both conditions manifest through the gradual degeneration of photoreceptors, with AMD predominantly impairing central vision and sparing peripheral vision, while RP impairs peripheral vision in the initial stages of the disease sparing central vision. Both conditions are marked by a significant decline in life quality for the patient (Flaxman et al. 2017; Wong et al. 2014; Bourne et al. 2021) . Historically, retinal prosthesis has been one of the medical strategies aimed at restoring some degree of vision for patients affected by these degenerative ocular diseases (Palanker and Goetz 2018; Lorach and Palanker 2017; Kien et al. 2012; Cheng et al. 2017).

So far, the successful retinal prostheses are designed to electrically stimulate the remaining intact inner retinal neurons, essentially replacing the function of the degenerated photoreceptors (PR) by direct activation of the retinal ganglion cells (RGC) and/or bipolar cells using large arrays of miniaturized electrodes (Kusumanchi et al. 2024; Hadjinicolaou et al. 2012; Hadjinicolaou et al. 2015; Sekirnjak et al. 2008). Traditional retinal implant designs predominantly utilize 2D planar electrodes, which, while demonstrating some success, present inherent limitations. For effective stimulation, the electrodes need to be in close proximity to the target retinal neurons. Given the flattened structure of 2D electrodes, achieving this close cellular proximity across the retina is challenging, often leading to non-uniform and imprecise stimulation and challenges with the spatial resolution (Flores et al. 2018). Compared to their planar counterparts, 3D electrodes allow cells to migrate and biomechanically interact with a larger surface area, potentially forming a network of neurons leading to more effective stimulation of the target cells (Bosi et al. 2015; Ho et al. 2019).

The investigation of 3D micropillar electrodes for retinal implants has resulted in promising improvements in visual acuity (Flores et al. 2018). The 3D geometry and small dimensions allow these electrodes to penetrate deeper into the retinal layers, thus establishing a closer and more consistent proximity to the target neurons. This may potentially lead to more precise and uniform stimulation, mimicking the natural, high-resolution functionality of healthy retinas. Closer proximity could also potentially lead to a more adequate stimulation of the retina, giving higher visual sensation for the same electrical or optical input. We recently proposed 3D pyrolytic carbon electrodes for use in retinal implants and demonstrated pre-testing without tissue, showing promising electrical properties of micropillars for retinal stimulation (Davidsen et al. 2019). The fabrication of such 3D structures through UV photolithography and pyrolysis offers a unique opportunity to test new and unexplored 3D architectures. The accurate adjustment of the 3D electrode width, height, and pitch e.g. to match the size of a single cell (~ 10 μm) is arguably more feasible for the pyrolytic carbon electrodes presented here compared to other methods for 3D electrode fabrication, since the two required fabrication steps allow for extreme dimensional control. Additionally, the fabrication of such pyrolytic carbon electrodes provides an advantage from a material perspective as it does not require any rare or expensive metals (Wang et al. 2005). Commonly used electrode materials such as iridium oxide (IrO_x_), often in the form of sputtered iridium oxide films (SIROF), platinum and gold are expensive and to some degree scarce (Ayton et al. 2020).

The established fabrication capabilities of 3D carbon electrodes (Hassan et al. 2017) and the known long lifetime of other medical implants using pyrolytic carbon (Salkeld et al. 2016; Vivas et al. 2022; PesÁkovÁ et al. 2000; Hassan et al. 2016) have motivated us to carry out ex vivo electrophysiological experiments with freshly dissected porcine neuroretinas. Electrophysiology experiments are pivotal in the development and optimization of retinal implants, serving as a crucial bridge between device/electrode designs and clinical applications (Wang et al. 2012). The experiments involve the recording of spiking activity of retinal cells such as RGC, bipolar cells, and amacrine cells in response to electrical stimuli.

We propose 3D pyrolytic carbon electrodes as an alternative for electrical retinal implants in general and the promising photovoltaic (PV) implants specifically. Therefore, in this paper, we investigate the possibilities of stimulating porcine retinal tissue with 2D and 3D pyrolytic carbon electrodes and analyze whether a minimum threshold voltage for increased spiking exists. By studying the effect of stimulation voltage on spike amplitude and comparing with spontaneous activity we also obtain information about the compound action potentials produced. This information could prove useful for anyone within the field fabricating devices for electrical stimulation of the retina.

## Materials and methods

### Microfabrication of pyrolytic carbon electrodes

#### Electrode design

**Fig. 1:**
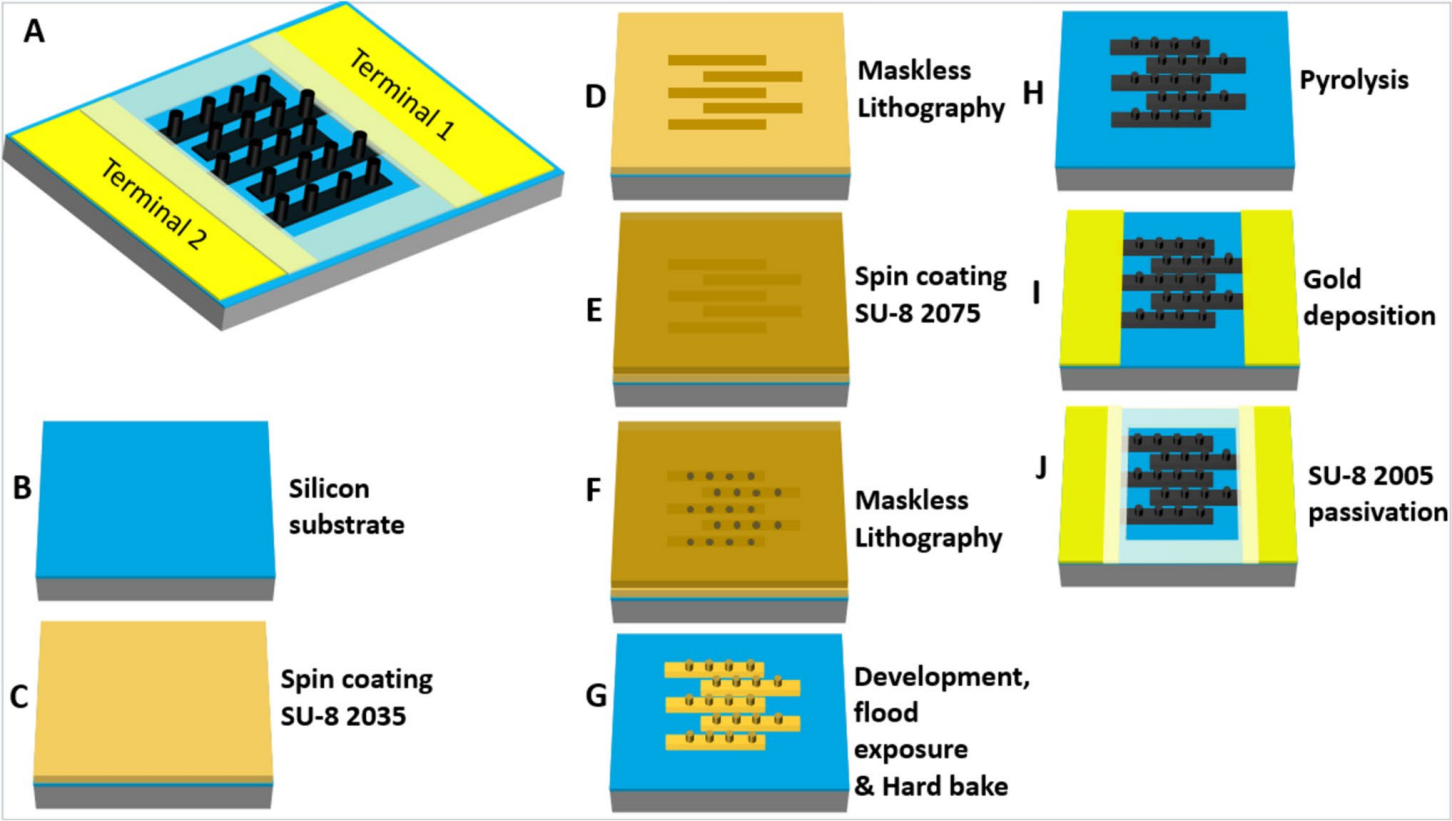
Design and fabrication process of the 3D interdigitated carbon electrodes. (**A**) Illustration of the 3D electrode design with carbon micropillars on each finger. (**B**) substrate preparation with thermally grown silicon oxide with thickness of 600 ± 20 nm; (**C-D**) spin coating of 15 μm SU-8 2035 with subsequent soft bake and a maskless lithography process to obtain 2D precursor structures; (**E**) spin coating of a 72 μm thick layer of SU-8 2075, and soft bake; (**F**) maskless lithography for definition of the pillar patterns; (**G**) development, flood exposure and hard bake; (**H**) carbon structures after pyrolysis; (**I**) E-beam deposition of Au contact pads; (**J**) SU-8 2005 passivation to define the ROI

For the electrophysiological studies, an interdigitated electrode (IDE) design with two independent terminals was chosen. Each terminal comprises 21 fingers, one of the terminals acting as stimulating electrode and the other terminal as local return electrode. The IDE design is symmetric, i.e. the stimulating and return electrode areas are identical. The IDE, as shown in Fig. 1A, is designed to have closely packed carbon electrode fingers with a width of 40 μm and a center-to-center pitch of 50 μm. On each finger, 3D carbon micropillars with a height of 30 μm, a width of 16 μm and a pitch of 50 μm are defined. The dimensions of the pillars are selected to be close to the size of typical cells (~ 10 μm) suitable for proper cell-electrode interaction (Flores et al. 2018; Ho et al. 2019). The SiO_2_ layer on the Si carrier substrate provides the necessary electrical isolation between the carbon electrode terminals. Larger Au pads are deposited at the end of the fingers as contacts for the electrical stimulation. To define the region of interest (ROI) for electrochemistry and electrophysiology, i.e. the area that is accessible for interaction with the retinal tissue, a passivation layer is designed.

#### Photoresist spin coating and patterning

Four-inch, n-type, single-side polished, 525 ± 20 μm thick Si wafers with < 100 > orientation were used as starting substrates. The general process of electrode fabrication has been described in previous work (Hassan et al. 2017). Figure1B-J shows the main steps of the fabrication. A 600 ± 20 nm SiO_2_ layer was thermally grown for 1 h at 1100 °C using a Tempress horizontal furnace with water vapor (15 standard litre per minute (slm)) as source for oxidation (Fig. 1B). Afterwards, a 20 min annealing was conducted with N_2_ (gas flow of 6 slm) in the same furnace. Prior to the process of spin coating, SU-8 photoresist (Kayaku AM Inc., Japan) was transferred into syringes and stored for > 12 h to eliminate any air bubbles. SU-8 is an epoxy based negative tone photoresist (Keller et al. 2008). The spin coating was carried out in a RCD8 spin coater from Süss MicroTec. SU-8 2035 photoresist was spin coated with a spreading step at 1000 rpm for 10 s and a final spin at 5000 rpm for 120 s to achieve a thickness of 15 μm (Fig. 1C). The resist was dried via soft bake at 50 °C for 15 min on a programmable hotplate and patterned using a maskless lithography process (MLA100, Heidelberg Instruments) with a wavelength of 365 nm and a dose of 270 mJ/cm^2^ with zero defocus (Fig. 1D). This step was succeeded by a post-exposure bake (PEB) on a hotplate for 2 h at 50 °C. For the patterning of the 3D micropillars, the process was continued with spin coating of SU-8 2075, first with a spreading step at 1000 rpm for 30 s, followed by a final spin at 2700 rpm for 60 s to obtain a total film thickness of approximately 72 μm (Fig. 1E). A soft bake was then carried out at 50 °C for 5 h. Another maskless lithography process was performed for patterning of the second layer as sketched in Fig. 1F, with a dose of 300 mJ/cm^2^ and zero defocus. A PEB for 2 h at 50 °C on a hotplate was performed to crosslink the exposed regions. For the 2D design the steps shown in Fig. 1E-F were omitted.

#### Development and hardening

After the lithography, the wafers were developed in two consecutive baths of propylene glycol monomethyl ether acetate (PGMEA) to remove SU-8 in the unexposed areas as shown in Fig. 1G followed by rinsing with isopropanol (IPA) and drying. Development time was 2 × 5 min for the 2D and 2 × 15 min for the 3D design. After drying, a flood exposure at a wavelength of 365 nm (Süss MicroTec Mask Aligner MA6) for 90 s with 11 mW/cm^2^ intensity and a hard bake on a hotplate for 15 h at 90 °C were done to further enhance the crosslinking of the photoresist.

#### Pyrolysis and gold deposition

The 2D and 3D SU-8 structures subsequently underwent pyrolysis in a multipurpose annealing furnace from ATV Technology GmbH conducted in N₂ atmosphere (20 slm). The thermal process protocol encompassed three distinct phases: Temperature was first increased with 5 °C/min to an initial hold at 375 °C for 30 min allowing for degassing of the photoresist and evaporation of non-carbon compounds. This was followed by a 3.33 °C/min ramping to reach 575 °C and a hold for 30 min at this temperature to promote carbonization (Hassan et al. 2017). The process continued with a 6 °C/min ramp to 1050 °C where the temperature was held for 3 h to obtain pyrolytic carbon as shown in Fig. 1H. After the heating, a steady cooling process was carried out at 10 °C/min. For proper Ohmic contacts two Au contact pads were deposited as shown in Fig. 1I. A steel shadow mask was designed and patterned with laser micromachining (microSTRUCT, 3D-Micromac AG). The shadow mask was placed on top of the device wafer and metal deposition was done by e-beam evaporation (Temescal, Ferrotec Co.) of 30 nm Cr at a rate of 1 Å/s and 200 nm Au at a rate of 5 Å/s rate.

#### Passivation layer

First, the wafer was treated in plasma with 170 ml/min O_2_ at 500 W for 2 min to scrub any residues that could lead to short circuits between the fingers. The definition of the ROI with the passivation layer was done by spin coating SU-8 2005 in RCD8 (Süss MicroTec) at 2000 rpm for 30 s, resulting in a 5 μm SU-8 layer. Maskless lithography (MLA100, Heidelberg Instruments) at 365 nm with a dose of 400 mJ/cm^2^ exposed the photoresist in the targeted pattern. The passivation layer, depicted in Fig. 1J, was developed by sequential immersion in two PGMEA baths for 1 min. This was followed by exposure at 365 nm UV light (Süss MicroTec Mask Aligner MA6) at 11 mW/cm² for 90 s, and then hardening through a bake at 90 °C for 15 h.

### Electrode characterization

**Fig. 2:**
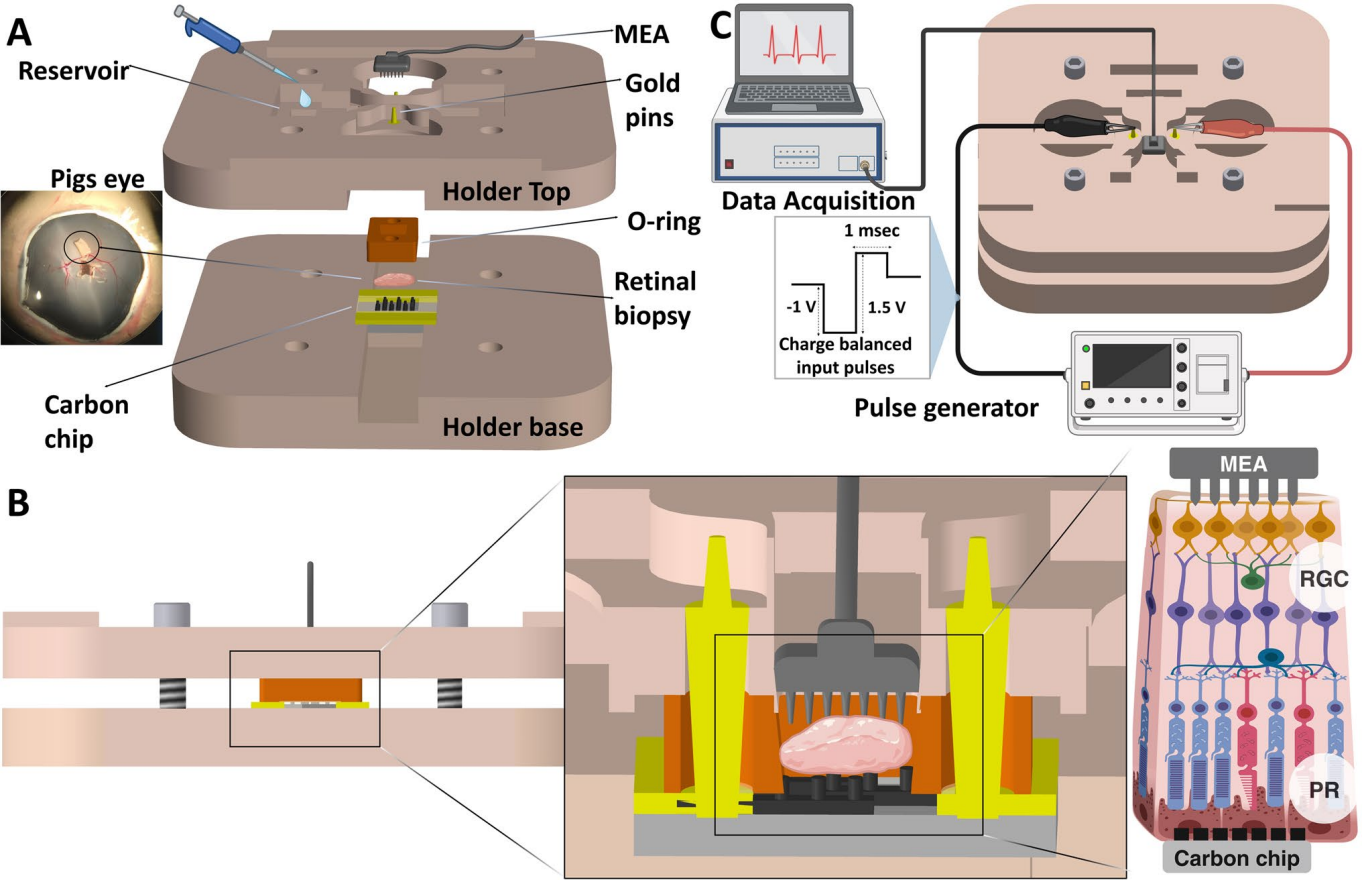
Experimental setup for electrophysiological measurements. (**A**) Exploded view of the 3D-printed holder assembly. The retinal tissue was placed upon the carbon electrodes on the chip and secured inside the holder by placing the 3D printed O-ring. The MEA was directed towards the tissue from the top of the reservoir. (**B**) Orientation of the tissue in the holder after assembly with the RGC facing the MEA while the carbon electrode chip was placed on the photoreceptor side. (**C**) The complete electrophysiological setup was placed in a Faraday cage with the holder assembly and the MEA inserted from the top. The Au pins on the holder were connected to the pulse generator. Data was recorded using a 32 channel Blackrock MEA connected to a Cereplex data acquisition unit (DAQ) via a Cereplex M headstage and stored on a PC. Image created with Biorender and Solidworks

First, cyclic voltammetry (CV) was used to confirm the functionality of the carbon electrodes and most importantly, to verify that the two IDE terminals were independent. Before the CV measurements, the electrodes were conditioned in an air plasma for 75 s (Pankratova et al. 2022). All CV experiments were carried out using a PalmSens4 potentiostat (PalmSens BV, The Netherlands). The chip with carbon electrodes was placed in a 3D printed holder, similar to the one shown in Fig. 2A but without the tissue and connected to the potentiostat with the instrument cell cable (PalmSens BV, The Netherlands). CV was conducted in 1 mL 10 mM K_3_[Fe(CN)_6_]/K_4_[Fe(CN)_6_] redox probe with external Pt counter electrode (CE) and a Ag/AgCl reference electrode (RE). The carbon electrodes were first connected with separate and then with connected terminals as the working electrode (WE). The scan window was set to −0.6 V to 1 V with a scan rate of 100 mV/s, and the experiment was repeated for 3 different chips from each design. For the estimation of the charge storage capacity (CSC) of the pyrolytic carbon electrodes, CV was measured in 1 mL Phosphate Buffered Saline (PBS: pH 7.4) using a scan window of −0.2 to 0.4 V, scan rate of 50 mV/s and an Ag/AgCl reference electrode. Here the stimulating electrode was used as WE and the other one as local CE. The scan rate of 50 mV/s was chosen based on literature (Cisnal et al. 2019; Nimbalkar et al. 2018; Boehler et al. 2020; Lipus and Krukiewicz 2022). From the cyclic voltammograms, the CSC is calculated according to Eq. (1) (Lipus and Krukiewicz 2022; Cisnal et al. 2019),

1 $$\:\text{C}\text{S}\text{C}=\frac1{Sv}\int_{E_1}^{E_2}I\left(E\right)\:\text{d}E$$

where *S* is the surface area in cm^2^ and $\:v$ is the scan rate, *E*_*1*_ and *E*_*2*_ are the cutoff potentials, and *I* is the current.

The geometrical area of the 3D electrode, derived from the original design and SEM images, included the dimensions of the fingers and pillars. For the 2D electrode, the area was based solely on the finger dimensions. The CSC calculation was averaged from four different 2D and 3D chips.

### Electrophysiological measurements

#### Chemical solutions

For storage and transportation, physiological saline solution (PSS) with the following composition was used: 118 NaCl mM, 4.8 KCl mM, 1.14 MgSO_4_ mM, 25 NaHCO_3_ mM, 5 mM Hepes, 1.5 mM CaCl_2_, 5.5 mM glucose. For experiments, PSS1.6 with the following composition was used: 119 NaCl mM, 4.7 KCl mM, 1.17 MgSO_4_ mM, 25 NaHCO_3_ mM, 5 mM Hepes, 1.6 mM CaCl_2_, 5.5 mM glucose, 1.18 mM KH_2_PO_4_, 0.026 mM EDTA. PSS0.0 refers to PSS1.6 where CaCl_2_ has been omitted. PSS1.6 was heated to 37°C and oxygenated by bubbling with a gas mixture of 95% atmospheric air and 5% CO_2_ prior to all experiments, to maintain the tissue at close to physiological conditions during electrical measurements. Tetrodotoxin (TTX) citrate was obtained from Hello Bio Reagents (Bristol, United Kingdom, HB1035). TTX solutions were prepared by preparing a stock solution in PSS1.6 of 1.0 M concentration. From this solution 1 mL aliquots of 2.25 mM concentration were prepared for use in future experiments. All TTX-solutions were stored at −20 °C between experiments.

#### Neuroretinal tissue preparation

Eyes from Danish Land Race pigs (*Sus domesticus*) were collected at a slaughterhouse (Danish Crown, Horsens, Denmark) immediately after the animals had been stunned with CO_2_ and euthanized by exsanguination. The eyes were transported to AUH laboratory in PSS at 4°C, and the time from the collection of the eyes to the commencement of the dissection procedure never exceeded 1 h. Dissection was performed in 4°C PSS0.0 as follows: Each eye was bisected at the equator with a double-edged razor blade, and the anterior segment was removed. The posterior segment containing the optical disk was placed under a stereo microscope, the vitreous body was removed. 2–3 mm from the optical disk a segment of approximately 3 mm x 10 mm neuroretina tissue was cut out using a self-locking chisel blade handle (VWR International, Herlev, Denmark) equipped with a 30 microblade (BD Beaver, D.J. Instruments, Billerica, USA). Care was taken to avoid large blood vessels in the segment selected.

#### Electrophysiology setup and stimulation

For electrophysiological experiments, a 3D printed holder was designed and printed with a Formlabs 3 printer. This holder was designed to accommodate the chip with carbon electrodes in the holder base. The tissue samples were placed on the chip with the photoreceptor layer facing down towards the carbon pillar electrodes. A silicone O-ring was placed on top of the chip, with the purpose of sealing the well inside the holder. PSS1.6 was then added to the reservoir as shown in Fig. 2A-B. The holder is designed to allow the MEA to be inserted from the top and moved down until it reaches the bottom of the holder top as shown in Fig. 2B. The tapered design of the O-ring prevents further movement of the MEA avoiding crashing into the carbon pillars. When tightened, the O-ring is compressed, creating an estimated gap of $\:\sim\:$ 500 ± 100 μm between the tip of the MEA electrode and the tip of the carbon electrode pillars.

A 32 channel Blackrock MEA was positioned on the RGC side of the tissue through the well after the lid was fastened to the holder bottom as shown in Fig. 2B-C. Also shown in Fig. 2C, a Keysight True Waveform Generator (33500B) was connected to the metal pins in the holder lid, providing contact to the Au pads of the chip and supplying electrical stimulation to the tissue. Data was recorded using the 32 channel Blackrock MEA connected to a Cereplex data acquisition unit (DAQ) via a Cereplex M headstage. Data was sampled at 30 kHz and high pass filtered at 250 Hz before being stored for further analysis.

To test whether the 2D and 3D electrodes could produce evoked biological responses above spontaneous activity, a series of stimulation experiments were carried out. First, a recording without stimulation was performed as a baseline indicative of spontaneous neuroretinal activity. The tissue samples were then stimulated by 1 ms pulses of 1 V amplitude, over a −500 mV offset, to provide a net amplitude of + 500 mV as shown in Fig. 2C. The negative offset was applied to avoid positive charge build up. Each stimulation run lasted for 10 s with stimulation at 10 Hz resulting in a total of 100 stimulations. To test whether recorded spikes were biological in origin, PSS 1.6 solution containing 5 µM TTX was added to the well after the first stimulation run within the setup (Lalonde et al. 2006; Chu et al. 2008). TTX is a voltage-gated Na^+^ channel antagonist and will prevent cells in the retina from generating action potentials. After administration of TTX, the tissue was allowed to rest for 5 min, and a second stimulation run with the same parameters as described previously was conducted. Six eyes from different animals were used for testing both the 2D and 3D electrodes.

To evaluate if there was a minimum threshold for the stimulation voltage, above which the carbon electrodes reliably induced evoked potentials in the tissue above spontaneous activity, a threshold voltage experiment was conducted. Tissue was stimulated at increasing voltages, starting at 0 V to get a baseline of spontaneous activity, then increasing by 100 mV steps until 1 V, where each stimulation step again consisted of stimulation with 1 ms pulses for 10 s at 10 Hz. After stimulation at 1 V, a new recording of spontaneous activity at 0 V was done, proceeded by two additional recordings at 500 and 1500 mV. After each stimulation the tissue was allowed 1 min of rest.

#### Data Analysis

Data analysis was performed using MatLab. The high pass filtered data traces were parsed into vectors by identifying the position of stimulation artefacts (SA) throughout the traces. As each stimulation run of 10 s used 10 Hz stimulation and since the data was sampled at 30 kHz, this yielded 100 vectors of 3000 datapoints, each starting with a SA. To avoid complications in data analysis caused by the SA at the beginning of each vector, and possibly at the end due to initial effects of the next SA, the vectors were truncated to only contain datapoints between 300 and 2700 post the initial SA.

Furthermore, to be counted as a spike, the points had to be a factor of 4.5 above the baseline noise for each recording. Evoked compound action potentials (spikes) were counted by identifying values in the truncated vectors which were more than a factor of 4.5 times above the baseline noise, defined as the root mean square (RMS) value of that entire vector. Furthermore, to avoid overcounting due to several datapoints of a single spike surpassing this value, a minimum of 30 datapoints (1 ms) had to pass before a new value could be counted as a spike. The criterion of 30 datapoints was based on numerous observations of the duration of evoked spikes. Evoked spikes tended to have returned to baseline after < 30 points, regardless of stimulation voltage.

This method of quantification had some consequences for the final spike counts. Firstly, the margin of 1 ms may cause some spikes to be discarded during high intensity spiking (buzzing), and potentially exceptionally large spikes may be counted more than once, if the duration surpasses 1 ms and amplitudes are still above 4.5 RMS. Secondly, since each data vector uses its own RMS level to determine spike counts, vectors with high spiking activity will tend to have higher RMS levels, resulting in a higher absolute exclusion criterion and thus, fewer spikes being counted. However, as the same procedure was applied to all recordings in the data analysis, the results are comparable, and the counting error should thus be similar. During some stimulation runs, some MEA channels would show no spiking, while others showed clear spiking. Examples of raw data traces from the same stimulation run, but from different channels, one spiking the other not, can be seen in Support Information (Sup. Figure 3). In all experiments involving TTX all channels were always included, as a non-spiking channel could be caused by the effects of TTX. For threshold voltage experiments, non-spiking channels were removed from further analysis to give clearer indications of spiking intensity where tissue was actively spiking. When spikes had been counted for all channels, the mean spike count for all channels with identifiable spikes was divided by the time span of the stimulation run to yield a mean spike count/sec for each stimulation run. For the estimation of the number of contributing neurons to the compound, the area under spikes was calculated using trapezoidal numerical integration with the ‘trapz’ function in Matlab. In order to include both the positive and the negative phase of given spike, the trapz function was performed on the absolute data for each spike. The baseline for the area under curve (AUC) estimations was 0.

#### Statistics

Data from the 2D and 3D electrode trials were evaluated using paired t-tests. Data from the threshold voltage experiment was analyzed using a one-way Kruskal Wallis ANOVA to test for significant effects of increasing voltage, followed by a Dunn’s post hoc testing to determine at which points significance occurred.

## Results

### Microfabrication of pyrolytic carbon electrodes

**Fig. 3:**
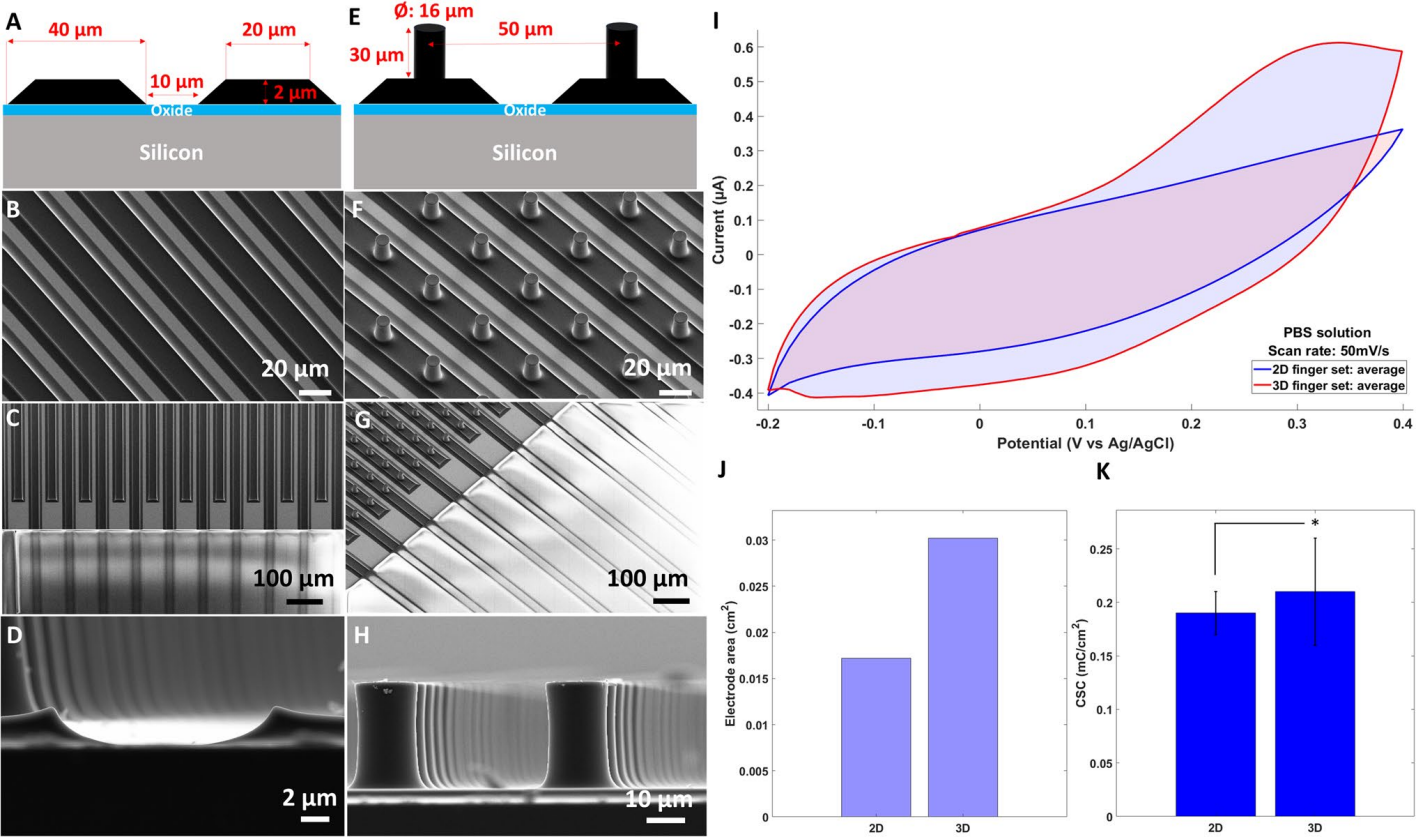
Pyrolytic carbon electrode characterization: (**A**) Schematic illustration of the dimensions of the 2D carbon IDE; (**B-D**) SEM images of the 2D carbon electrodes showing (**B**) the independent fingers and (**C**) a passivation layer on the fingers defining the boundary for the ROI. (**D**) The close-up view at the carbon finger edges visualizes the shrinkage during pyrolysis; (**E**) Dimensions of the 3D carbon electrode design with micropillars on top of 2D carbon fingers; (**F-H**) SEM images of carbon pillars on the independent carbon fingers, with (**G**) the passivation layer defining the ROI. (**H**) The close-up view of the micropillars shows a narrower pillar diameter at the center and a larger diameter at the top and bottom; (**I**) Comparative analysis of a single IDE terminal of 2D and 3D electrodes with CV recorded in PBS solution with a scan rate of 50 mV/s. The shaded areas indicate capacitive currents integrated for extraction of the charge storage capacity (CSC); (**J**) estimated electrode surface area for 2D and 3D designs and (**K**) corresponding CSC in mC/cm^2^

The fabrication process was optimized to obtain pyrolytic carbon electrodes with the dimensions specified for the 2D and 3D designs. The evaluation of the final dimensions was done after pyrolysis to obtain accurate values for the finger width and the pillar height and diameter. Figure 3A illustrates the dimensions and shape of the carbon fingers with bottom widths of 40 ± 3 μm corresponding to the original design, while the top width was reduced to 20 ± 1 μm due to the lateral shrinkage during pyrolysis. The final thickness of the carbon fingers was 2 ± 0.75 μm. Figure 3B shows the 2D IDE after completed fabrication. The passivation layer defines the boundary for the carbon region accessible for electrostimulation and indicates the ROI for the retinal tissue in Fig. 3C. Figure 3B and D confirm a trapezoidal cross-section of the 2D carbon layer after the pyrolysis process due to the shrinkage. Figure 3E presents the dimensions of the microfabricated carbon pillars with heights of 30 μm and diameters of 16 μm, arranged with a center-to-center spacing (pitch) of 50 μm. Figure 3F show the pillar arrangement on the interdigitated fingers indicating the alignment accuracy of the lithography process and presenting the passivation layer defining the boundary of the ROI. The magnified SEM image of the pillars in Fig. 3H shows that the pyrolysis process caused minor vertical variance in pillar diameter, resulting in a diameter of 16 ± 1 μm at the pillar top compared to 15 ± 0.5 μm at half the height.

The functionality of the IDE was confirmed through CV with a redox probe for both 2D and 3D configurations, as detailed in the supplementary information. The fact that peak currents in cyclic voltammograms recorded for the individual terminals were approximately 50% compared to the ones for the two combined terminals (Sup. Figure 2) confirmed the absence of short circuits between the electrode fingers.

### Extraction of the charge storage capacity of the pyrolytic carbon electrode material

CV was performed and the CSC was calculated according to Eq. (1). Figure 3I shows the cyclic voltammograms obtained with single terminals of the 2D and 3D pyrolytic carbon IDE in PBS solution with a scan rate of 50 mV/s. The areas enclosed by the CV curves represent the capacitive behavior of the electrodes, showing that the capacitance of the 3D electrodes was higher than the one of the 2D design. The electrode area of the 2D and 3D electrodes calculated by considering the number and dimensions of the fingers and micropillars was 0.0172 cm^2^ and 0.0302 cm^2^, respectively (Fig. 3J). With the values of the surface area the CSC was calculated. Figure 3(k) shows a CSC of 0.21 ± 0.05 mC/cm² for 3D and a CSC of 0.19 ± 0.02 mC/cm² for 2D electrodes. The fact that the CSC was similar for both electrode designs indicates that the porosity and the electrical properties of the pyrolytic carbon material in general seemed to be independent of the electrode geometry evaluated in this study.

### Electrostimulation of porcine neuroretinas with 3D carbon micropillar electrodes

**Fig. 4:**
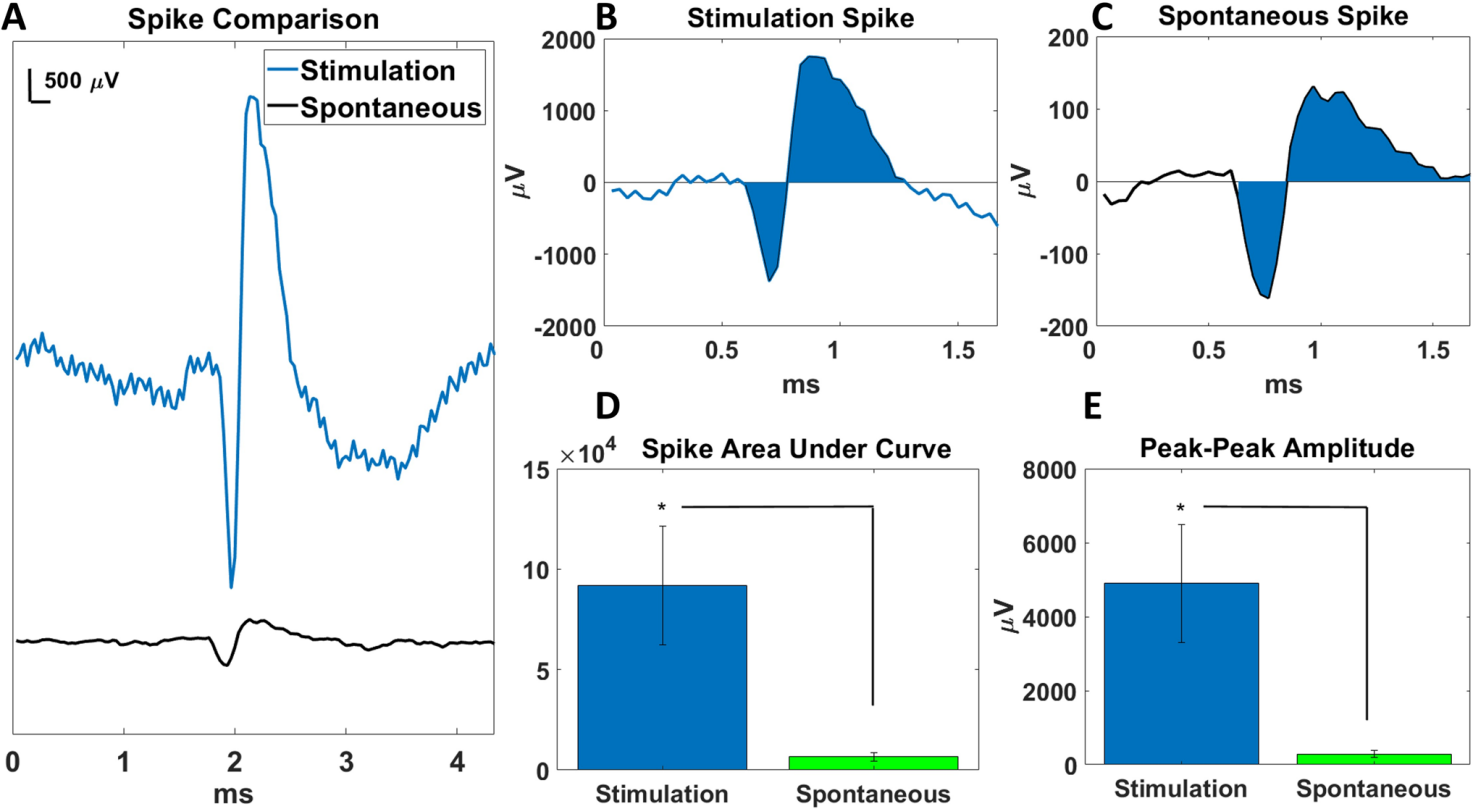
Comparison of single spike waveforms and area under curve. (**A**) The blue trace is evoked with a 1 ms pulse of 500 mV and the black trace is spontaneous. (**B**, **C**) Close up of the evoked and spontaneous spikes with indication of the area under curve. (**D**) Area under curve, found by trapezoidal numerical integration, for all identified spikes evoked from six retinas using 1 ms 500 mV pulses stimulating at 10 Hz. (**E**) Peak-peak amplitude of all identified spikes evoked from six retinas using 1 ms 500 mV pulses stimulating at 10 Hz. The significant difference is marked as * p ≤ 0.05

The primary goals of this study were to establish whether 3D pyrolytic carbon micropillar electrodes can stimulate the neuroretina and produce spiking, and whether 3D electrodes offer an advantage over 2D. For this purpose, MEA measurements of stimulated porcine neuroretinas were conducted and analyzed. Figure 4 shows representative examples of single stimulated and spontaneous compound action potentials, as well as a comparison of the area under curve for evoked and spontaneous counted spikes. As can be seen in this example, the waveform and duration of the two action potentials is relatively similar (Fig. 4A, B,C). However, there is an order of magnitude difference in peak-to-peak amplitude for evoked spikes (> 3 mV), compared to spontaneous spikes ($\:\sim\:$200 µV). This holds true for all identified spikes as well (Fig. 4E), where there was a significant difference (*p* < 0.05) between the amplitudes of evoked and spontaneous spikes. Likewise, there was a significant difference in the area under curve for evoked and spontaneous spikes (*p* < 0.05). The comparison of all counted spikes in Fig. 4D shows an average increase in the area under curve for evoked spikes by a factor of approximately 14 compared to spontaneous activity.

**Fig. 5:**
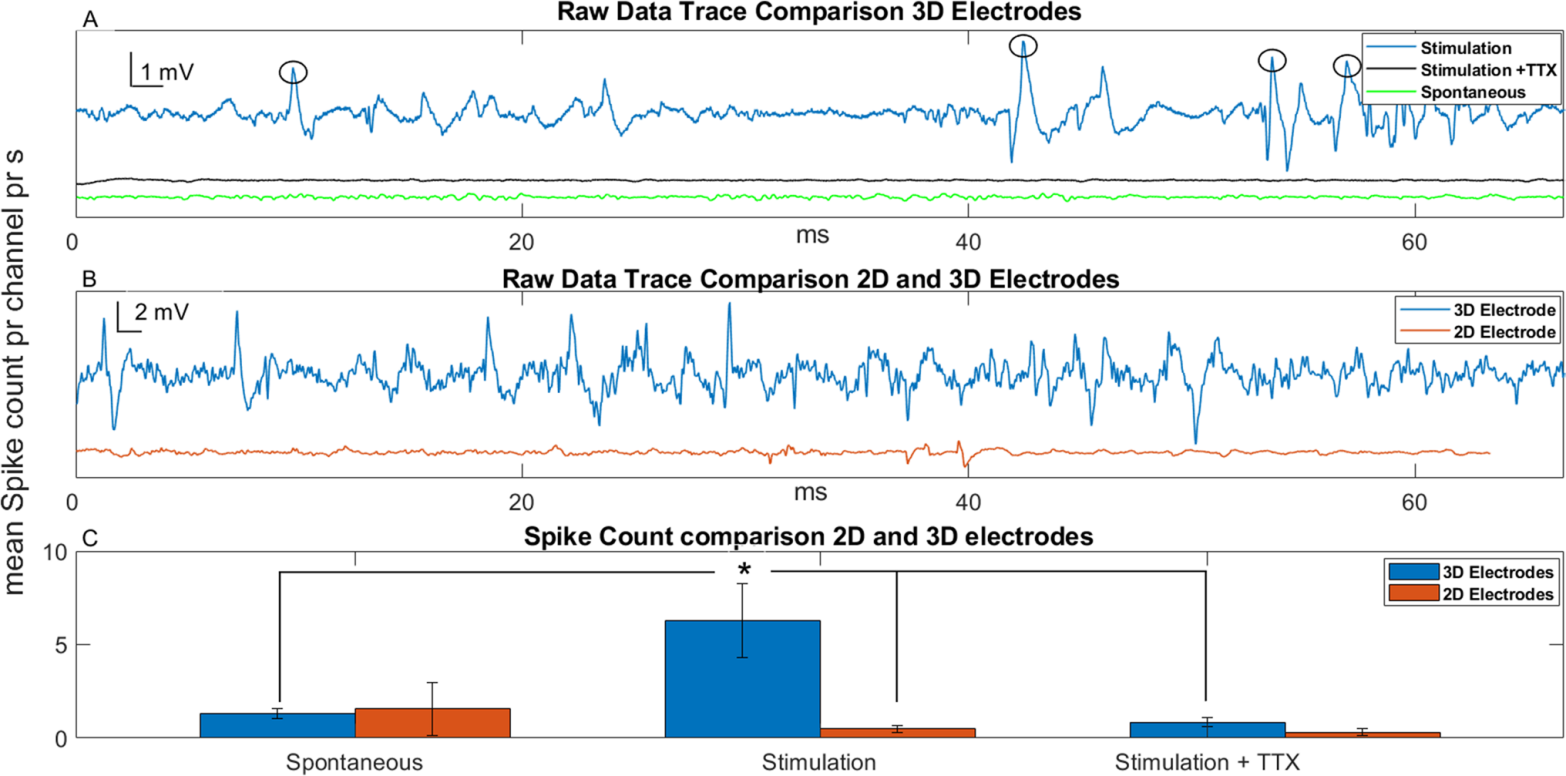
Electrophysiological recordings with 2D and 3D pyrolytic carbon electrodes. (**A**) Examples of raw data traces recorded during electrical stimulation of porcine retina. Pulses were 1 ms at 10 Hz in all cases. Spontaneous activity (green), stimulation at 500 mV (blue) and stimulation at 500 mV after exposure to TTX (black), black circles indicate spikes which were counted above the 4.5 x RMS threshold for the stimulation trace. (**B**) Example raw traces of stimulation via 3D (blue) and 2D (red) electrodes at 500 mV. (**C**) Results from electrophysiological recordings, 3D electrode data in blue, 2D electrode data in red. Mean spike count/channel/s for 500 mV stimulation for 3D electrodes, 2D electrodes, both with and without added TTX, and spontaneous data from both types of electrodes. For all data types n = 6, bars indicate standard errors. The significant difference is marked as * p ≤ 0.05

Data from stimulation of six different neuroretinas are presented in Fig. 5. Figure 5A shows examples of raw data traces for spontaneous retinal activity recorded prior to stimulation and for neuroretinas stimulated with the 3D pyrolytic carbon micropillar electrodes. Furthermore, the stimulated trace recorded after exposure to TTX is included. There is a clear increase in spiking in the traces with applied electrical stimulation, both in number of spikes and their amplitude compared to spontaneous neural activity, indicating the successful activation of retinal cells with the electrodes. This increase in spike rate and amplitude is eliminated by the addition of TTX, confirming the biological nature of the recorded signals. Figure 5B shows examples of raw traces from stimulation via 3D and 2D electrodes, respectively. The traces show higher spike rate and larger spike amplitudes for 3D electrodes. The quantified results of these stimulation experiments for all six retinas are shown in Fig. 5C. Figure 5C includes the results for stimulation using both 3D and 2D electrodes, with their respective TTX controls, along with spontaneous activity for tissue placed on both 3D and 2D electrodes. There was a significant increase in the spike count/channel/s for tissue stimulated with the 3D electrodes compared to the corresponding spontaneous activity (*p* < 0.05). Subsequent exposure of the neuroretina to TTX significantly reduced the spike count during electrical stimulation with 3D electrodes (*p* < 0.05).

For 2D electrodes, differences between spontaneous, stimulated activity and stimulated activity after exposure to TTX were not significant (*p* = 0.2 and *p* = 0.23, respectively). Note, that the reason for higher mean spike counts for spontaneous activity compared with tissue stimulated with the 2D electrodes, is likely increased noise introduced by connecting the waveform generator to the setup. When spontaneous activity was measured, no generator was connected, allowing low amplitude spontaneous spikes to be recorded. These spikes were not recordable when the generator was connected for active stimulation. Finally, 3D electrodes produced significantly higher spike counts than 2D electrodes upon electrical stimulation (*p* < 0.05) (Fig. 5B-C).

### Influence of the voltage on retinal stimulation with 3D electrodes

**Fig. 6:**
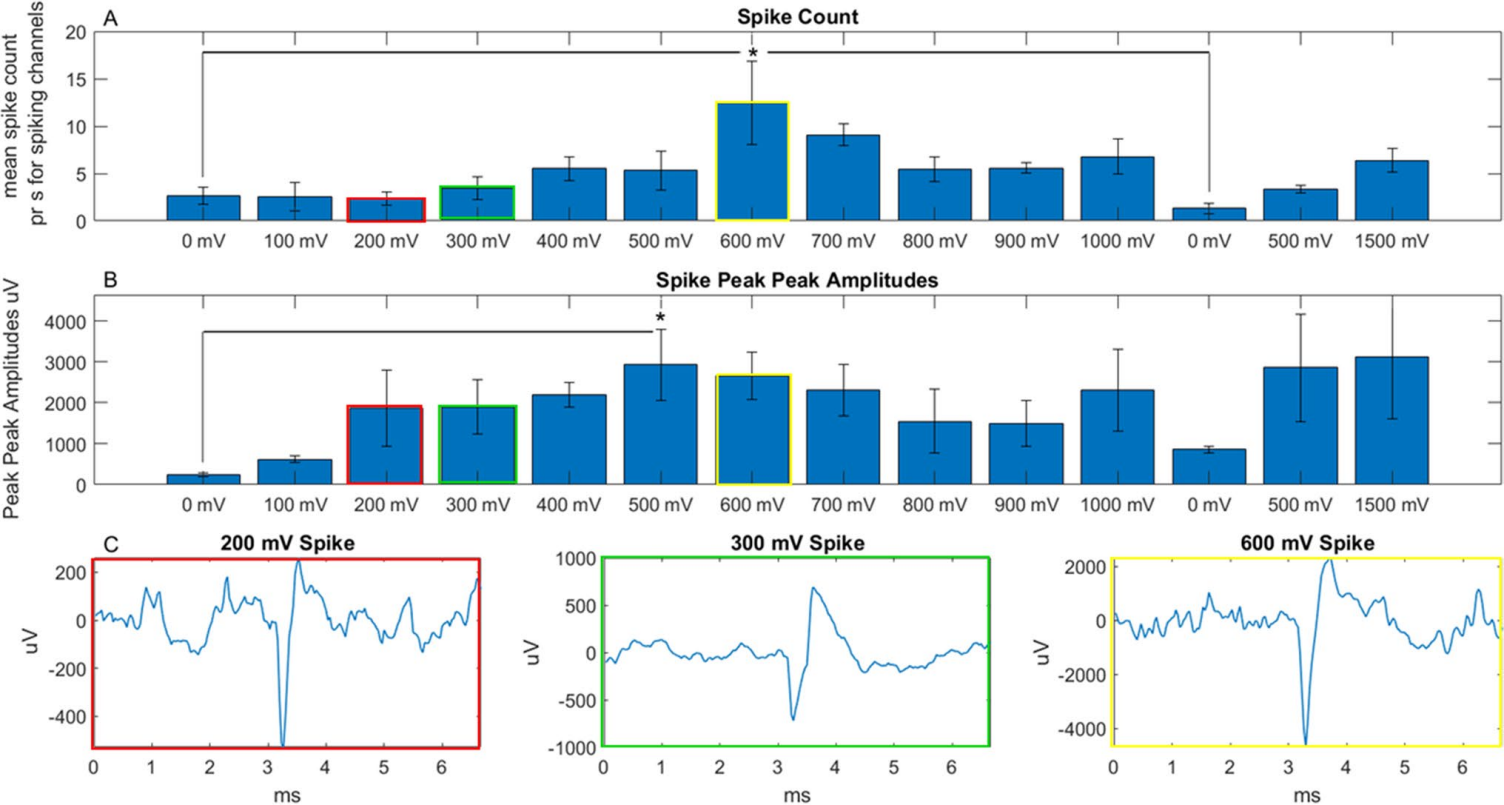
Investigation of the influence of stimulation voltage on neuroretina activation. (**A**) 3D electrode threshold test spike count data. Mean spike count/s for spiking channels through 100 mV increments, 0 mV (spontaneous activity) to 1000 mV, followed by recordings again at 0 mV, then stimulation again at 500 mV and finally stimulation at 1500 mV. (**B**) Peak-peak amplitude of spikes measured in (A), through stimulation steps as described above. For both sets of data, *n* = 6, bars indicate standard error. (**C**) Examples of single spikes at 200, 300 and 500 mV stimulation, respectively. The significant difference is marked as * *p* ≤ 0.05

The secondary goal of this study was to investigate how the evoked biological activity depends on stimulation voltage and if there is a threshold voltage that is required to induce compound action potentials, and how the amplitude of these potentials depended on stimulation voltage. Due to the discovered superior performance of the 3D electrodes at 1 ms stimulation duration, the ramp test was carried out using the 3D electrodes only. Figure 6 shows data from stimulation of six neuroretinas for the experiment with increasing stimulation voltages. Figure 6A shows spike counts obtained throughout the incremental increase of voltage during the test. Note that only channels showing clear spiking activity were included. This differs from the results presented in Fig. 4, where all channels were included. One-way ANOVA confirmed that spike counts increased with increasing voltage (*p* < 0.05). Subsequent post hoc testing showed that the spike counts for 600 mV stimulation was significantly different from both the initial and the second 0 mV spike counts.

Figure 6A indicates that spike counts for 100–400 mV were comparable to mean spontaneous activity (spike rate at 0 mV). Following this peak at 600 mV, spike counts drop of and do remain lower for the rest of the test, even at 1500 mV.

Figure 6B shows peak-peak amplitudes of the spikes recorded at different stimulation voltages. Here also, one-way ANOVA showed significant increase in amplitude as voltage increased (*p* < 0.05). Unlike the spike counts shown in Fig. 6A, there appears to be a gradual increase in peak-peak amplitudes as stimulation voltage increases, peaking initially at 500 mV. There was significant difference between the initial 0 mV amplitudes and those recorded at 500 mV. After returning to 0 mV the highest amplitudes were recorded at 1500 mV stimulation. The comparison of isolated spikes in Fig. 6C shows that as voltage increases and amplitudes increase, there is no corresponding increase in the duration of the spikes.

## Discussion

For stimulating neural electrodes, thin film metal electrodes such as IrOx, Pt, and Au are typically used for retinal implants due to their electrical properties. Among these, IrOx stands out with a significantly higher charge storage capacity (CSC) of approximately 182 ± 30 mC/cm^2^ (Maeng et al. 2020), markedly surpassing Pt (12.4 ± 0.8 mC/cm^2^) and Au (2.2 ± 0.1 mC/cm^2^) (Lipus and Krukiewicz 2022). This underlines the superior electrochemical performance of IrOx, facilitating enhanced stimulation capabilities. For comparison, the pyrolytic carbon electrodes in this work exhibit a much lower CSC of 0.21 ± 0.05 mC/cm^2^, aligning closely with that of glassy carbon (GC) electrodes at 0.25 mC/cm^2^ (Nimbalkar et al. 2018). The relatively low CSC is attributed to the fact that pyrolytic carbon obtained from photoresist precursors has a very low surface roughness and porosity (Hassan et al. 2017). Surface roughness has been presented as a method to increase CSC drastically, e.g. for PEDOT-PSS coated ITO electrodes (Yang et al. 2015). Despite this apparent limitation in charge storage, the appeal of pyrolytic carbon electrodes lies in their exceptional thermal stability, durability, cost-effectiveness, and biocompatibility (Behzadi et al. 2012). These characteristics, coupled with the fabrication flexibility allowing for precise dimensional tailoring, render pyrolytic carbon a viable option for stimulating electrodes. The capability of pyrolytic carbon electrodes to effectively stimulate neurons, despite their lower CSC, indicates an excellent contact between electrode and tissue. In addition, it highlights a trade-off between electrochemical performance and material properties such as stability, cost, and fabrication versatility in the design and selection of neural stimulating electrodes.

The first question of this study was whether pyrolytic carbon electrodes are able to stimulate neuroretinal tissue electrically, and if so, to which extent. Also, it was investigated whether 3D electrodes perform better than 2D. Figure 5shows the results of electrical stimulation via carbon electrodes for porcine retinas from six different eyes, stimulated with 500 mV pulses of 1 ms duration at a frequency of 10 Hz, with both 2D and 3D electrodes. We deliberately chose a relatively short pulse duration of 1 ms, which is on par with the shortest pulses typically used for e.g. photovoltaic retinal stimulation (Palanker and Goetz 2018; Lorach and Palanker 2017; Ho et al. 2020). The stimulation charge increases with the pulse duration (Sekirnjak et al. 2006), and minimizing the duration ensures a lower applied charge and thereby current. With this, any effects observed at given voltages are not enhanced by an unreasonably high current. The electrical stimulation using 3D electrodes resulted in a significant increase of spiking activity compared to spontaneous activity without stimulation. The measured increase in mean spike rate during stimulation with 3D electrodes over spontaneous activity reported in Fig. 5 is approximately three-fold. For 2D electrodes, no significant difference between spontaneous and stimulated spiking activity was observed. The resulting spikes observed in the traces of Fig. 5A and B were greatly reduced or even completely removed when adding TTX to the medium containing the tissue, for both 2D and 3D electrodes. The inhibition of the spiking activity of retinal cells using TTX has been reported by several groups such as Hood (1999), Frishman (2000) and Hare (2002). Specifically in porcine models, Lalonde et al. (Lalonde et al. 2006; Chu et al. 2008) reported disappearance of multifocal electroretinogram spike components P2 and P3 and reduced amplitude of P1. Therefore, it can be concluded that the observed spikes in Fig. 5A in our study are very likely biological in nature and dependent on voltage-gated Na^+^ channel activity i.e. they are compound action potentials generated by the retinal cells. The results clearly demonstrate that active stimulation of the neuroretina with the investigated electrical pulses was achieved with 3D pyrolytic micropillar electrodes while 2D electrodes did not produce any increase in activity.

The significant difference between the two electrode geometries, in light of the wide-spread and well-accepted use of planar electrodes, calls for a discussion of the underlying cause. Since the 2D and 3D pyrolytic carbon electrodes were fabricated with the exact same material using identical processes, we may attribute any observed differences to the geometry of the electrodes. While the positive effect of 3D electrodes on e.g. subretinal photovoltaic implants (Flores et al. 2018; Ho et al. 2019) is well-known, it was not previously clear whether such an effect would also be pronounced for non-PV electrical stimulation. It was also unknown whether an improved effect of 3D electrodes would be identified for pyrolytic carbon, as it has only been investigated for other electrode materials such as iridium oxide (SIROF), Au- and Pt-coated Si pillars and pure metal. Finally, while the positive effect is well-known, the cause of the significant difference has only been partly discussed in the case of PV retinal implants. The typical explanation given is the effect of cell attraction to cavities on the surface, which results in closer proximity of cells to stimulating electrodes, compared to planar devices. While we fully agree with this explanation, we hypothesize whether the previously reported favorable cell adhesion to pyrolytic carbon pillars (Amato et al. 2014) could also explain the positive neural response achieved with these 3D electrodes. Another factor to consider is the actual electrode configuration used in our measurements where an interdigitated format was used with one terminal serving as the stimulating WE and the other one as the return CE. This configuration was chosen because it more closely corresponds to PV retinal implant designs with local return electrodes. In our study, the 2D carbon IDE have a thickness of only 2 μm. For an applied potential, electrical field lines between WE and CE terminals will mostly run in parallel and close to the underlying substrate. This would limit the volumetric interaction of the electric field to a few µm into the retinal tissue on top of the electrodes. For 3D pillar electrodes, the situation is different and field lines between WE and CE will be more radially distributed, interacting with deeper layers of the retinal tissue. This could further explain the successful neural stimulation achieved with the 3D electrode geometry. A more in-depth investigation of the electrical field distribution requires theoretical modeling, and we will investigate this further in future work. We note that the resistive loss in the specific saline medium (PSS) between stimulating electrodes and tissue influences the voltage drop from electrode to neuron. As such, an accurate estimate of the electric field intensity at the site of stimulation could be made via e.g. finite-element modelling by comparing the output voltages reported here, with the expected biological threshold voltage at the surface of a retinal neuron (e.g. a bipolar cell) and then factoring in the effect of an intermediate medium with a resistivity that can be verified experimentally for the PSS used here. Such detailed simulation was outside the scope of this work but will be investigated in future work. Goo et al. measured porcine RGC responses from current pulses of 0.5–2 ms duration (Cha et al. 2021) and found that a degenerated porcine retina requires higher charge than the healthy control. This confirms the importance of investigating current and voltage thresholds for electrical stimulation of the retina. Therefore, we investigated the effect of increasing stimulation voltage on spike count and peak-peak amplitude. The data in Fig. 6 shows a significant increase in both spike rate and amplitude with increasing stimulus voltage. The point at which evoked responses increased significantly above spontaneous tissue activity occurred in the 500 mV – 600 mV range. The increasing amplitudes also initially peak at 500 mV. The amplitude data shows that there is an approximately 12-fold increase between the amplitude of spikes evoked by stimulation at + 500 mV compared to the amplitude of spontaneous spiking.

The large increase in amplitude upon electrical stimulation is a significant finding. As we are measuring compound action potentials produced by the retinal cells, likely a combination of RGC, amacrine cells, and bipolar cells, the increase in amplitude indicates stimulation of multiple cells responding close to simultaneously. Figure 4 suggests that the waveforms of evoked and spontaneous spikes are very similar, the main difference being the amplitude of the spikes. This suggests that the increase in spike amplitude during 3D electrode stimulation, is caused by more cells firing simultaneously, while to a smaller extent by cells increasing their firing rate.

According to Schalow et al. (Schalow et al. 1995) the number of contributing cells may be estimated, to a first approximation, by comparing the area under the curve of the single and compound action potentials, respectively. This assumes linear superposition of the individual action potentials as described by Stegeman (Stegeman and De Weerd 1982) and others. Therefore, the approximately 14-fold increase in area under curve observed in Fig. 4D implies an increase of the amount of firing cells for evoked spikes of approximately a factor of 14 compared to spontaneous spikes. We acknowledge that this value is a rough estimate, since it relies heavily on the accuracy of the numerical integration. Yet, it indicates a realistic size of the compound neuron population, which in this case is ~ 14 times larger than spontaneous activity.

The reason for no further significant increase in spike rate or amplitude beyond 700 mV is unknown. It may be the case that all viable cells are responding at this point, and thus no further increase occurs. It may also be the case, that as the voltage is continuously increased, the cells in the tissue are depleted or deteriorating, preventing a further increase of the stimulation responses. Other studies (Hopper et al. 2023) have reported an asymptotic behavior of the compound action potential amplitude as electrical stimulus is increased.

To our knowledge, a voltage-threshold of 500–600 mV for porcine retinal stimulation considering compound action potentials evoked by carbon electrodes has not been reported before. Rathbun et al.(Jalligampala et al. 2017) reported anodic and cathodic voltage and spike duration thresholds for wild-type (*wt*) and *rd10 *mice. They found an optimal voltage-controlled pulse of −2.4 V and 0.88 ms. For the 1 ms spike duration in (Jalligampala et al. 2017) an apparent anodic threshold of ~ 1.6 V is seen, while for cathodic voltages spike rates seem to increase above − 0.7 V. Ye et al. similarly recorded RGC spiking from *rd10 *mice and found increased spiking at a voltage of 0.7 V, but estimated by sigmoidal fitting a threshold voltage of 0.55 V. Samba et al. (2015) compared TiN with PEDOT-CNT coated Au electrodes using rat retinas and measured threshold voltages (at 1 ms pulses) of 600 mV for TiN and 200 mV for PEDOT-CNT electrodes, respectively. Stett et al.(Stett et al. 2000) measured spike rates from isolated chicken retina at 0.8–1.6 V and found that at 0.8 V spike rates only increased when the electrode was very close to the tissue, while at 1.2 and 1.6 V spike rates increased even at ~ 100 μm distance. Gekeler et al. (2004) measured cortical activity from subretinal electrical stimulation of the rabbit retina and reported threshold voltages in the range 0.1–2.38 V, with an average of 0.94 V (*n*= 10). In comparison, the voltage threshold range of 0.6–0.7 V measured in our study is on par and generally slightly lower than the reported thresholds in (Jalligampala et al. 2017; Samba et al. 2015; Stett et al. 2000; Gekeler et al. 2004), except for the case of PEDOT-CNT coated electrodes stimulating rat retinas in (Samba et al. 2015). Whether these differences are due to differences between the pig model used here and the mouse/rat/chicken/rabbit models in (Jalligampala et al. 2017; Samba et al. 2015; Stett et al. 2000; Gekeler et al. 2004) and their retinal cell response, is unclear, but could be investigated further.

The threshold voltage range of 500–600 mV is particularly interesting for PV retinal implants, given their intrinsic properties of voltages dictated by the quality and number of PV diodes per pixel. As such, a good silicon-based single-diode pixel produces ~ 600 mV, while two diodes in series produce ~ 1200 mV. For this reason, the most efficient state-of-the-art PV implants (Palanker and Goetz 2018; Lorach and Palanker 2017; Flores et al. 2018; Ho et al. 2019) typically use two or even three diodes in series per pixel. While this is reasonable in terms of ensuring a sufficiently high stimulation voltage per pixel, it does put a limitation on the pixel density (per area) for a given PV implant. Our data on the effect of stimulation voltage (Fig. 6) confirms the need for at least 600 mV to achieve significant increase in count and amplitude of compound action potentials indicating effective neural stimulation. While such a voltage close to the practical limit of Si PV indicates the need for at least two diodes in series, we hypothesize that there is a small chance for effective stimulation with just a single Si diode. However, this would require excellent contact between electrodes and tissue, which properly designed 3D electrodes may provide. Furthermore, it would require optimized Si PV diodes with stimulation voltages as close to the limit of conventional Si PV as possible. This must be verified in a future study using PV implants with 3D carbon electrodes and varying number of diodes per pixel, for comparison.

Ideally, 3D electrodes in retinal implants should be designed ensuring that charge is only injected at the very tip of each pillar electrode. Therefore, the tips of typical pillar electrodes used in PV retinal implants are coated with a highly capacitive material like SIROF (Ho et al. 2019). Alternatively, the sidewalls could be selectively passivated by a biocompatible material, such that only the tip is exposed, thereby ensuring similar current confinement and localized charge injection from pillar tips. Rezaei et al. (2022) showed how such a sidewall passivation can be achieved on 3D carbon pillars using polydopamine passivation and tip opening with localized laser ablation. We hypothesize, that such a selective sidewall passivation may improve the performance of 3D carbon electrodes even further. This might not just increase the mean spike rate compared to planar electrodes, but also confine the charge injection to local spots and ideally minimize crosstalk between neighboring ‘pixels’ in a PV or electrical implant.

## Conclusion

This study investigates whether – and to which extent – 3D pyrolytic carbon electrodes are able to stimulate neuroretinas electrically, and whether 3D electrodes could outperform 2D electrodes. We fabricated carbon pillar electrodes by pyrolysis of various SU-8 structures defined photolithographically on Si wafers. Then, we measured electrical responses from porcine neuroretinas stimulated electrically with carbon electrodes. Our results clearly show that 3D electrodes induce significantly higher counts of compound action potentials in retinal cells than 2D. The biological nature of the recorded spikes was validated by using TTX as inhibitor confirming that the measured electrical signals were voltage-gated sodium channel dependent. Our hypothesis is that the significant difference between 2D and 3D electrodes is not only caused by geometrical benefits of 3D pillars, but also material-related properties of 3D carbon pillars, such as increased adhesion of the retinal tissue. Our findings indicate that 3D carbon pillar electrodes are promising for electrical stimulation of the retina.

We systematically investigated spike rate and spike amplitude as a function of stimulation voltage and found an apparent voltage threshold, above which the spike rate significantly increased compared to spontaneous activity. Our results indicate a threshold range of 500–600 mV for 1 ms pulses at a frequency of 10 Hz. Our results also indicate that the main increase in neural activity caused by electrical stimulation is expressed as increasing amplitudes of compound action potentials, i.e. activating more cells which fire simultaneously, and to a lesser extent increasing the firing rate of individual cells. Based on numerical integration we estimate the area under the curve to be ~ 14 times larger in evoked compound action potentials compared to spontaneous activity, presumably indicating the relative increase in number of cells contributing to the compound.

## Supplementary Information

Below is the link to the electronic supplementary material.

Supplementary file1 Micrographs of the carbon pillar device including passivation (SF-1), details of the C-V data (SF-2) and examples of raw traces from spiking and non-spiking channels (SF-3). (PDF 438 KB)

## Data Availability

No datasets were generated or analysed during the current study.
