# Supplementary material for: Electrical stimulation of neuroretinas with 3D pyrolytic carbon electrodes
Source: Biomed Microdevices. 2025 Feb 11;27(1):7. doi: 10.1007/s10544-024-00729-8 (PMC11813987; doi:10.1007/s10544-024-00729-8)
Supplement: Supplementary file 1 — Supplementary file1 Micrographs of the carbon pillar device including passivation (SF-1), details of the C-V data (SF-2) and examples of raw traces from spiking and non-spiking channels (SF-3). (PDF 438 KB) [file 10544_2024_729_MOESM1_ESM.pdf]

Supporting Information

The foundational electrode design, used for preliminary evaluation of the setup has one uniform base supporting the pillars as shown in Supporting Figure 1.

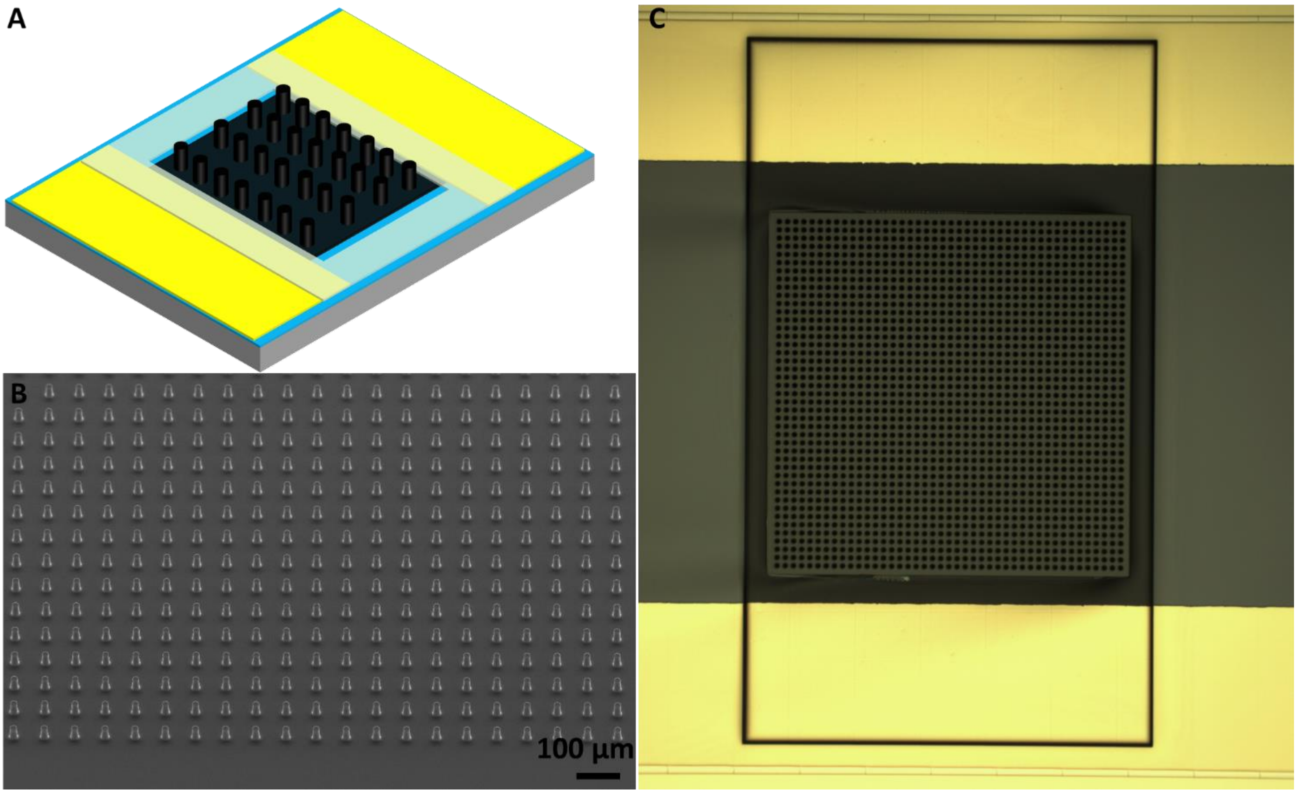

Supp. Figure 1: (A) 3D pyrolytic carbon electrode with pillars on one single base, (B) SEM image of the electrode showing the array of pillars. (C) Optical image of the device showing the passivation layer defining the ROI.

Interdigitated electrode functionality

The electrochemical characterization of the 2D and 3D design in redox probe (**Supp. Figure 2**) indicates the contribution of current from independent terminals vs external Pt counter, is lower than the resulting currents from both terminals connected and the peak-to-peak potential separation (ΔE_p) indicated the performance of the 2D and 3D design, explaining the fast electron transfer kinetics. Overall, the individuality of the fingers in the design is confirmed and the experiment is carried out for n=3 different chips for 2D and 3D.

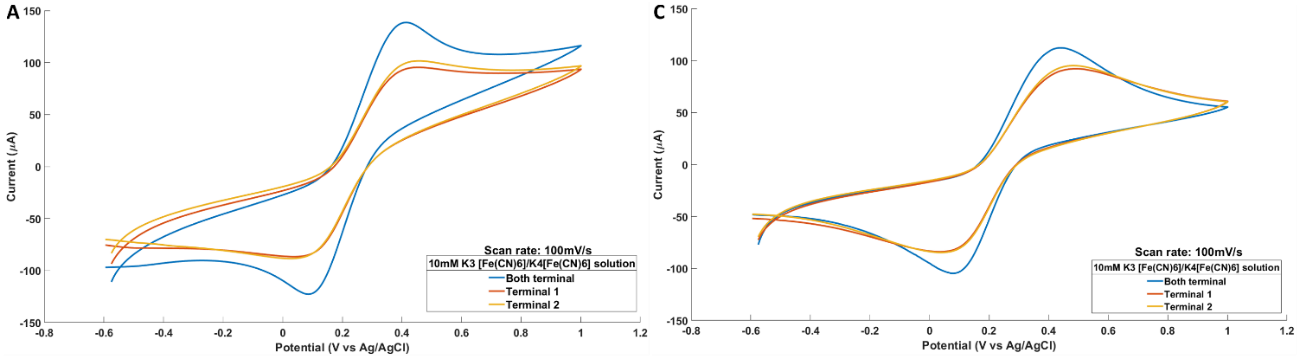

B

Working Electrode	I_{pa} (µA)	I_{pc} (µA)	ΔE_p (V)
Terminal 1	-79.60 ± 2.2	92.76 ± 2.4	0.34 ± 0.014
Terminal 2	-80.22 ± 2.8	93,7 ± 3.8	0.33 ± 0.012
Terminal 1&2	-123.2 ± 3.2	130 ± 6.6	0.29 ± 0.018

D

Working Electrode	I_{pa} (µA)	I_{pc} (µA)	ΔE_p (V)
Terminal 1	-76.33 ± 6.8	90.22 ± 4.7	0,38 ± 0.039
Terminal 2	-81.41 ± 5.5	91.3 ± 2.6	0.37 ± 0.056
Terminal 1&2	-118 ± 12.8	124 ± 7.4	0.34 ± 0.032

Supp. Figure 2: Electrochemical characterization of interdigitated electrodes with varying configurations: (A) Average data of the CVs for 2D design, (B) table for the ΔE_p, anodic and cathodic currents for the respective CV, (C) average CV data of the 3D design (D) ΔE_p, anodic and cathodic currents for the respective CV for each terminal configuration.

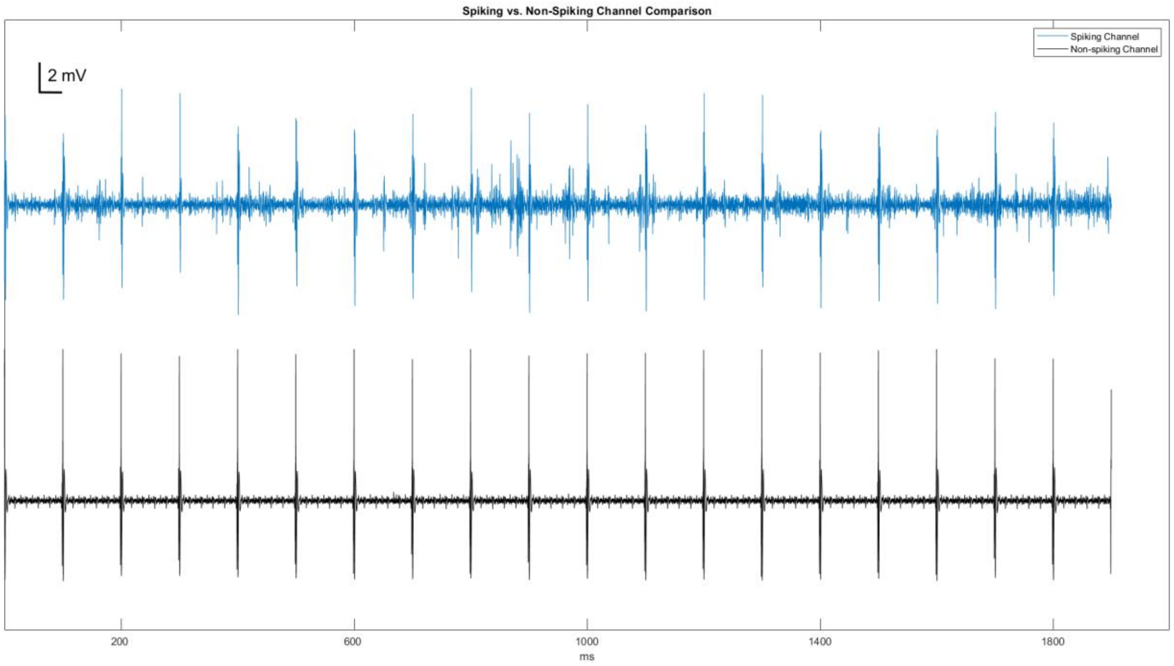

Supp. Figure 3: Examples of raw data traces from two different channels during stimulation with 500 mV in 1 ms pulses of the same tissue sample. One channel (blue) shows clear spiking, while the other (black) shows no spikes. For the spike count quantification, non-spiking channels such as shown here, were removed from the count, as to not undercount the number of evoked spikes, recorded in channels where the tissue was reactive.
